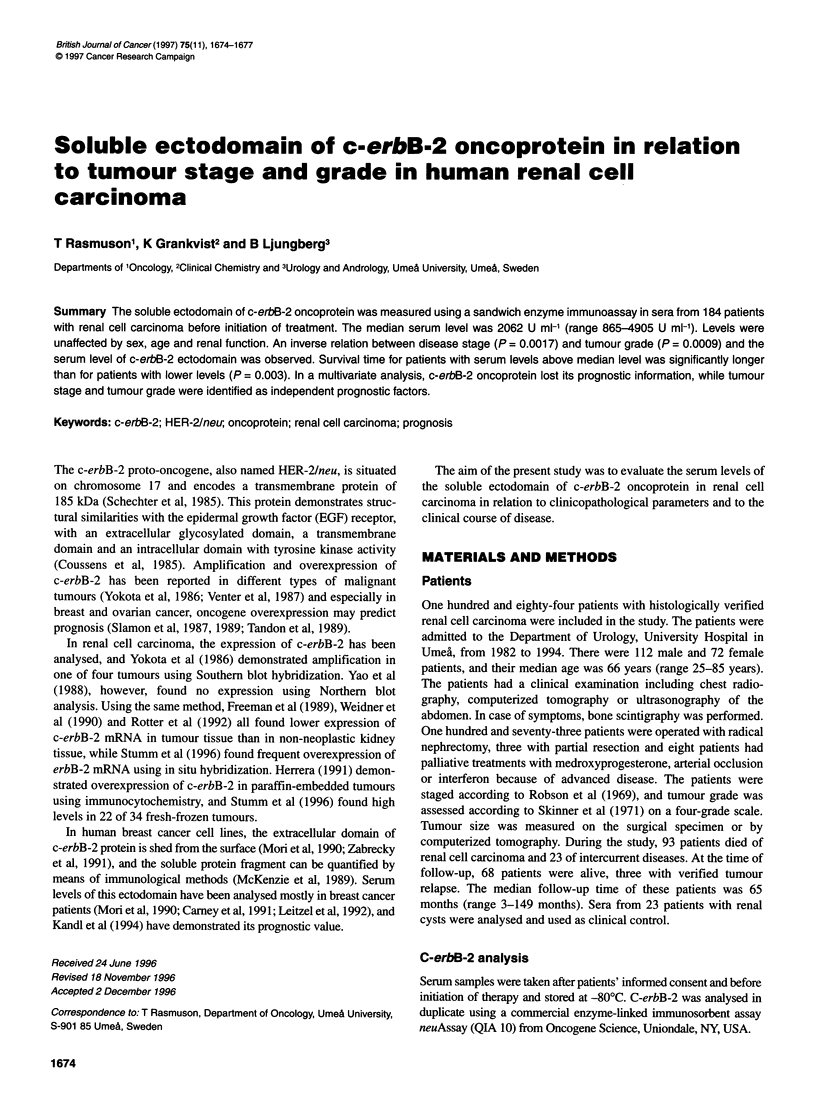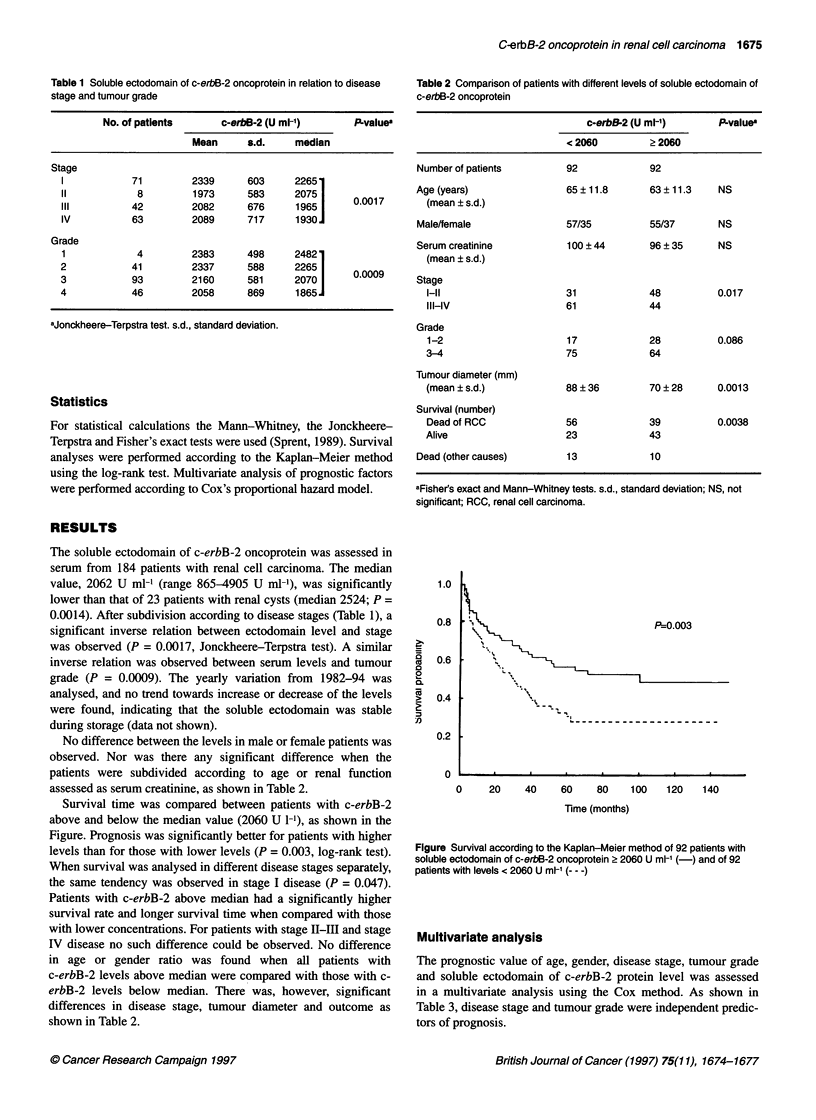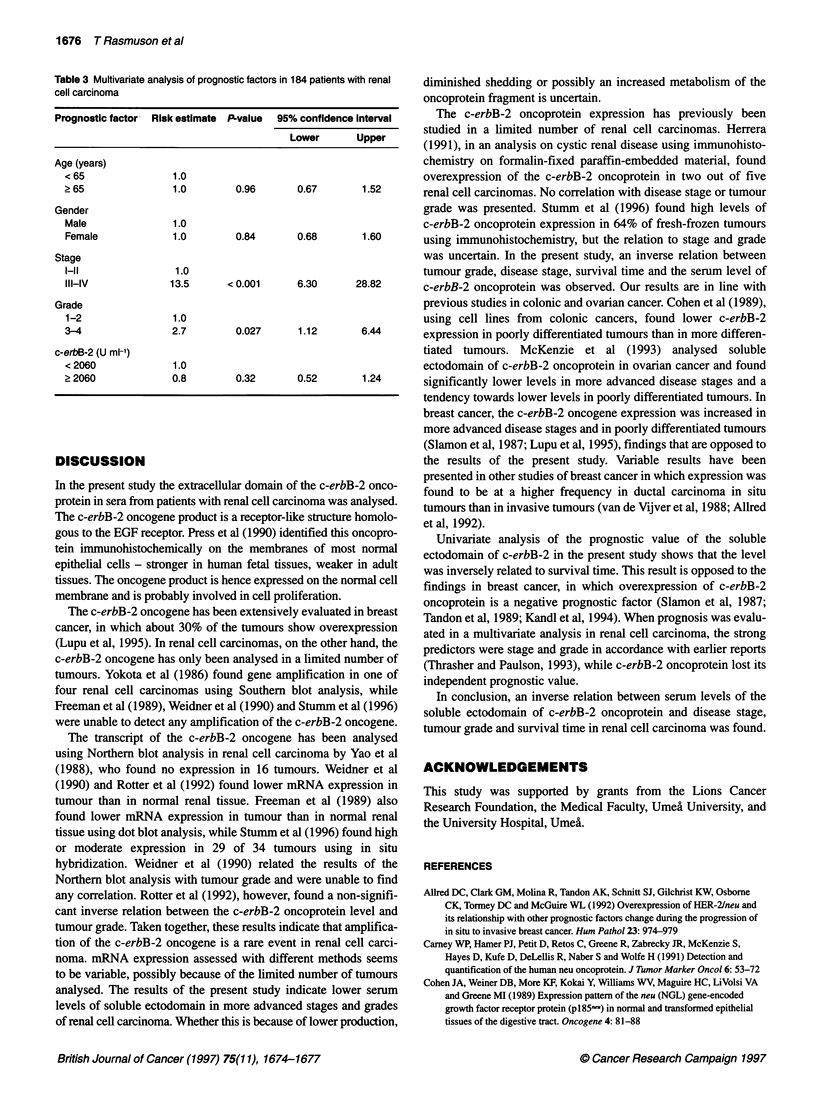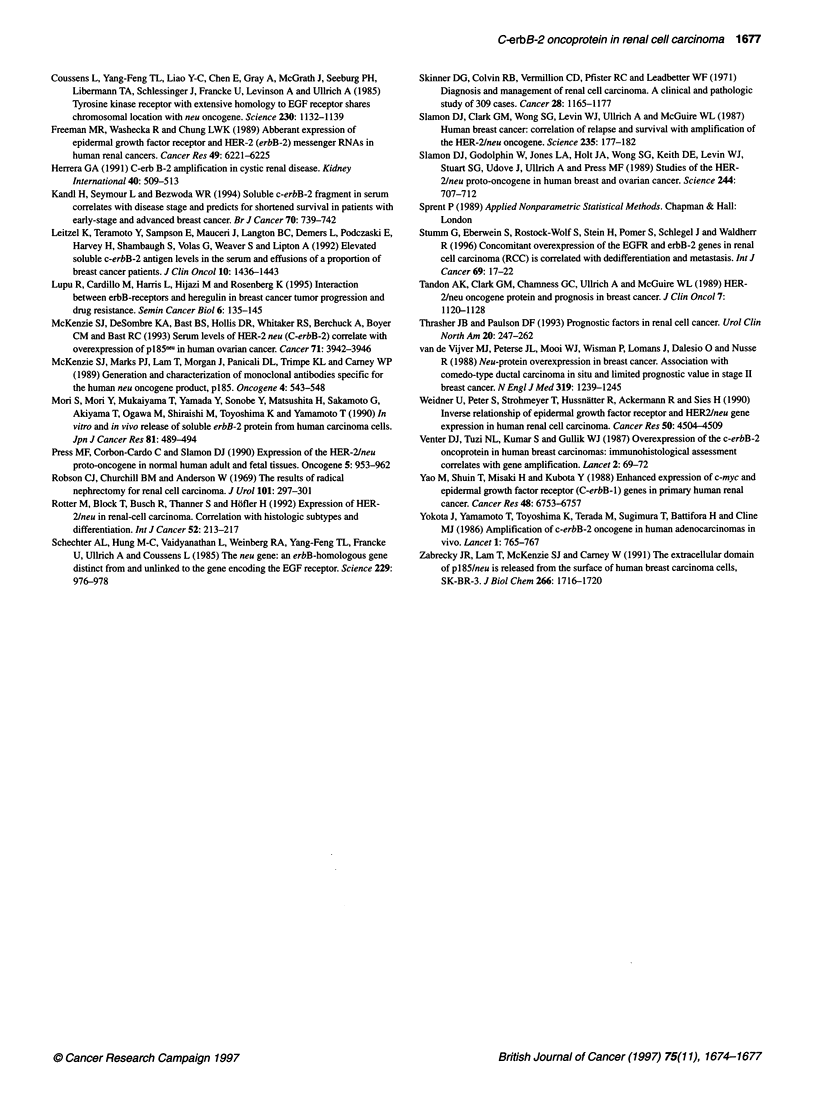# Soluble ectodomain of c-erbB-2 oncoprotein in relation to tumour stage and grade in human renal cell carcinoma.

**DOI:** 10.1038/bjc.1997.284

**Published:** 1997

**Authors:** T. Rasmuson, K. Grankvist, B. Ljungberg

**Affiliations:** Department of Oncology, UmeÃ¥ University, Sweden.

## Abstract

The soluble ectodomain of c-erbB-2 oncoprotein was measured using a sandwich enzyme immunoassay in sera from 184 patients with renal cell carcinoma before initiation of treatment. The median serum level was 2062 U ml(-1) (range 865-4905 U ml(-1)). Levels were unaffected by sex, age and renal function. An inverse relation between disease stage (P = 0.0017) and tumour grade (P = 0.0009) and the serum level of c-erbB-2 ectodomain was observed. Survival time for patients with serum levels above median level was significantly longer than for patients with lower levels (P = 0.003). In a multivariate analysis, c-erbB-2 oncoprotein lost its prognostic information, while tumour stage and tumour grade were identified as independent prognostic factors.


British Journal of Cancer (1997) 75(11), 1674–1677
© 1997 Cancer Research Campaign

### Soluble ectodomain of c-*erb*B-2 oncoprotein in relation to tumour stage and grade in human renal cell carcinoma

**T Rasmuson[1], K Grankvist[2] and B Ljungberg[3]**

Departments of [1]Oncology, [2]Clinical Chemistry and [3]Urology and Andrology, Umeå University, Umeå, Sweden

**Summary** The soluble ectodomain of c-*erb*B-2 oncoprotein was measured using a sandwich enzyme immunoassay in sera from 184 patients with renal cell carcinoma before initiation of treatment. The median serum level was 2062 U $ml^{-1}$ (range 865–4905 U $ml^{-1}$). Levels were unaffected by sex, age and renal function. An inverse relation between disease stage ($P = 0.0017$) and tumour grade ($P = 0.0009$) and the serum level of c-*erb*B-2 ectodomain was observed. Survival time for patients with serum levels above median level was significantly longer than for patients with lower levels ($P = 0.003$). In a multivariate analysis, c-*erb*B-2 oncoprotein lost its prognostic information, while tumour stage and tumour grade were identified as independent prognostic factors.

**Keywords:** c-*erb*B-2; HER-2/*neu*; oncoprotein; renal cell carcinoma; prognosis

The c-*erb*B-2 proto-oncogene, also named HER-2/*neu*, is situated on chromosome 17 and encodes a transmembrane protein of 185 kDa (Schechter et al, 1985). This protein demonstrates structural similarities with the epidermal growth factor (EGF) receptor, with an extracellular glycosylated domain, a transmembrane domain and an intracellular domain with tyrosine kinase activity (Coussens et al, 1985). Amplification and overexpression of c-*erb*B-2 has been reported in different types of malignant tumours (Yokota et al, 1986; Venter et al, 1987) and especially in breast and ovarian cancer, oncogene overexpression may predict prognosis (Slamon et al, 1987, 1989; Tandon et al, 1989).

In renal cell carcinoma, the expression of c-*erb*B-2 has been analysed, and Yokota et al (1986) demonstrated amplification in one of four tumours using Southern blot hybridization. Yao et al (1988), however, found no expression using Northern blot analysis. Using the same method, Freeman et al (1989), Weidner et al (1990) and Rotter et al (1992) all found lower expression of c-*erb*B-2 mRNA in tumour tissue than in non-neoplastic kidney tissue, while Stumm et al (1996) found frequent overexpression of *erb*B-2 mRNA using in situ hybridization. Herrera (1991) demonstrated overexpression of c-*erb*B-2 in paraffin-embedded tumours using immunocytochemistry, and Stumm et al (1996) found high levels in 22 of 34 fresh-frozen tumours.

In human breast cancer cell lines, the extracellular domain of c-*erb*B-2 protein is shed from the surface (Mori et al, 1990; Zabrecky et al, 1991), and the soluble protein fragment can be quantified by means of immunological methods (McKenzie et al, 1989). Serum levels of this ectodomain have been analysed mostly in breast cancer patients (Mori et al, 1990; Carney et al, 1991; Leitzel et al, 1992), and Kandl et al (1994) have demonstrated its prognostic value.

*Received 24 June 1996*
*Revised 18 November 1996*
*Accepted 2 December 1996*

*Correspondence to:* T Rasmuson, Department of Oncology, Umeå University, S-901 85 Umeå, Sweden

The aim of the present study was to evaluate the serum levels of the soluble ectodomain of c-*erb*B-2 oncoprotein in renal cell carcinoma in relation to clinicopathological parameters and to the clinical course of disease.

### MATERIALS AND METHODS

### Patients

One hundred and eighty-four patients with histologically verified renal cell carcinoma were included in the study. The patients were admitted to the Department of Urology, University Hospital in Umeå, from 1982 to 1994. There were 112 male and 72 female patients, and their median age was 66 years (range 25–85 years). The patients had a clinical examination including chest radiography, computerized tomography or ultrasonography of the abdomen. In case of symptoms, bone scintigraphy was performed. One hundred and seventy-three patients were operated with radical nephrectomy, three with partial resection and eight patients had palliative treatments with medroxyprogesterone, arterial occlusion or interferon because of advanced disease. The patients were staged according to Robson et al (1969), and tumour grade was assessed according to Skinner et al (1971) on a four-grade scale. Tumour size was measured on the surgical specimen or by computerized tomography. During the study, 93 patients died of renal cell carcinoma and 23 of intercurrent diseases. At the time of follow-up, 68 patients were alive, three with verified tumour relapse. The median follow-up time of these patients was 65 months (range 3–149 months). Sera from 23 patients with renal cysts were analysed and used as clinical control.

### C-*erb*B-2 analysis

Serum samples were taken after patients' informed consent and before initiation of therapy and stored at –80°C. C-*erb*B-2 was analysed in duplicate using a commercial enzyme-linked immunosorbent assay *neu*Assay (QIA 10) from Oncogene Science, Uniondale, NY, USA.

**Table 1** Soluble ectodomain of c-*erb*B-2 oncoprotein in relation to disease stage and tumour grade

| | No. of patients | c-*erb*B-2 (U ml$^{-1}$) Mean | s.d. | median | P-value[a] |
|---|---|---|---|---|---|
| Stage | | | | | |
| I | 71 | 2339 | 603 | 2265 | |
| II | 8 | 1973 | 583 | 2075 | 0.0017 |
| III | 42 | 2082 | 676 | 1965 | |
| IV | 63 | 2089 | 717 | 1930 | |
| Grade | | | | | |
| 1 | 4 | 2383 | 498 | 2482 | |
| 2 | 41 | 2337 | 588 | 2265 | 0.0009 |
| 3 | 93 | 2160 | 581 | 2070 | |
| 4 | 46 | 2058 | 869 | 1865 | |

[a]Jonckheere–Terpstra test. s.d., standard deviation.

### Statistics

For statistical calculations the Mann–Whitney, the Jonckheere–Terpstra and Fisher's exact tests were used (Sprent, 1989). Survival analyses were performed according to the Kaplan–Meier method using the log-rank test. Multivariate analysis of prognostic factors were performed according to Cox's proportional hazard model.

### RESULTS

The soluble ectodomain of c-*erb*B-2 oncoprotein was assessed in serum from 184 patients with renal cell carcinoma. The median value, 2062 U ml$^{-1}$ (range 865–4905 U ml$^{-1}$), was significantly lower than that of 23 patients with renal cysts (median 2524; $P$ = 0.0014). After subdivision according to disease stages (Table 1), a significant inverse relation between ectodomain level and stage was observed ($P$ = 0.0017, Jonckheere–Terpstra test). A similar inverse relation was observed between serum levels and tumour grade ($P$ = 0.0009). The yearly variation from 1982–94 was analysed, and no trend towards increase or decrease of the levels were found, indicating that the soluble ectodomain was stable during storage (data not shown).

No difference between the levels in male or female patients was observed. Nor was there any significant difference when the patients were subdivided according to age or renal function assessed as serum creatinine, as shown in Table 2.

Survival time was compared between patients with c-*erb*B-2 above and below the median value (2060 U l$^{-1}$), as shown in the Figure. Prognosis was significantly better for patients with higher levels than for those with lower levels ($P$ = 0.003, log-rank test). When survival was analysed in different disease stages separately, the same tendency was observed in stage I disease ($P$ = 0.047). Patients with c-*erb*B-2 above median had a significantly higher survival rate and longer survival time when compared with those with lower concentrations. For patients with stage II–III and stage IV disease no such difference could be observed. No difference in age or gender ratio was found when all patients with c-*erb*B-2 levels above median were compared with those with c-*erb*B-2 levels below median. There was, however, significant differences in disease stage, tumour diameter and outcome as shown in Table 2.

**Table 2** Comparison of patients with different levels of soluble ectodomain of c-*erb*B-2 oncoprotein

| | c-*erbB*-2 (U ml$^{-1}$) < 2060 | ≥ 2060 | P-value[a] |
|---|---|---|---|
| Number of patients | 92 | 92 | |
| Age (years) (mean ± s.d.) | 65 ± 11.8 | 63 ± 11.3 | NS |
| Male/female | 57/35 | 55/37 | NS |
| Serum creatinine (mean ± s.d.) | 100 ± 44 | 96 ± 35 | NS |
| Stage | | | |
| I–II | 31 | 48 | 0.017 |
| III–IV | 61 | 44 | |
| Grade | | | |
| 1–2 | 17 | 28 | 0.086 |
| 3–4 | 75 | 64 | |
| Tumour diameter (mm) (mean ± s.d.) | 88 ± 36 | 70 ± 28 | 0.0013 |
| Survival (number) | | | |
| Dead of RCC | 56 | 39 | 0.0038 |
| Alive | 23 | 43 | |
| Dead (other causes) | 13 | 10 | |

[a]Fisher's exact and Mann–Whitney tests. s.d., standard deviation; NS, not significant; RCC, renal cell carcinoma.

**Figure** Survival according to the Kaplan–Meier method of 92 patients with soluble ectodomain of c-*erb*B-2 oncoprotein ≥ 2060 U ml$^{-1}$ (—) and of 92 patients with levels < 2060 U ml$^{-1}$ (- - -)

### Multivariate analysis

The prognostic value of age, gender, disease stage, tumour grade and soluble ectodomain of c-*erb*B-2 protein level was assessed in a multivariate analysis using the Cox method. As shown in Table 3, disease stage and tumour grade were independent predictors of prognosis.

© Cancer Research Campaign 1997

**Table 3** Multivariate analysis of prognostic factors in 184 patients with renal cell carcinoma

| Prognostic factor | Risk estimate | P-value | 95% confidence interval | |
|---|---|---|---|---|
| | | | Lower | Upper |
| Age (years) | | | | |
| < 65 | 1.0 | | | |
| ≥ 65 | 1.0 | 0.96 | 0.67 | 1.52 |
| Gender | | | | |
| Male | 1.0 | | | |
| Female | 1.0 | 0.84 | 0.68 | 1.60 |
| Stage | | | | |
| I–II | 1.0 | | | |
| III–IV | 13.5 | < 0.001 | 6.30 | 28.82 |
| Grade | | | | |
| 1–2 | 1.0 | | | |
| 3–4 | 2.7 | 0.027 | 1.12 | 6.44 |
| c-*erb*B-2 (U $ml^{-1}$) | | | | |
| < 2060 | 1.0 | | | |
| ≥ 2060 | 0.8 | 0.32 | 0.52 | 1.24 |

### DISCUSSION

In the present study the extracellular domain of the c-*erb*B-2 oncoprotein in sera from patients with renal cell carcinoma was analysed. The c-*erb*B-2 oncogene product is a receptor-like structure homologous to the EGF receptor. Press et al (1990) identified this oncoprotein immunohistochemically on the membranes of most normal epithelial cells – stronger in human fetal tissues, weaker in adult tissues. The oncogene product is hence expressed on the normal cell membrane and is probably involved in cell proliferation.

The c-*erb*B-2 oncogene has been extensively evaluated in breast cancer, in which about 30% of the tumours show overexpression (Lupu et al, 1995). In renal cell carcinomas, on the other hand, the c-*erb*B-2 oncogene has only been analysed in a limited number of tumours. Yokota et al (1986) found gene amplification in one of four renal cell carcinomas using Southern blot analysis, while Freeman et al (1989), Weidner et al (1990) and Stumm et al (1996) were unable to detect any amplification of the c-*erb*B-2 oncogene.

The transcript of the c-*erb*B-2 oncogene has been analysed using Northern blot analysis in renal cell carcinoma by Yao et al (1988), who found no expression in 16 tumours. Weidner et al (1990) and Rotter et al (1992) found lower mRNA expression in tumour than in normal renal tissue. Freeman et al (1989) also found lower mRNA expression in tumour than in normal renal tissue using dot blot analysis, while Stumm et al (1996) found high or moderate expression in 29 of 34 tumours using in situ hybridization. Weidner et al (1990) related the results of the Northern blot analysis with tumour grade and were unable to find any correlation. Rotter et al (1992), however, found a non-significant inverse relation between the c-*erb*B-2 oncoprotein level and tumour grade. Taken together, these results indicate that amplification of the c-*erb*B-2 oncogene is a rare event in renal cell carcinoma. mRNA expression assessed with different methods seems to be variable, possibly because of the limited number of tumours analysed. The results of the present study indicate lower serum levels of soluble ectodomain in more advanced stages and grades of renal cell carcinoma. Whether this is because of lower production, diminished shedding or possibly an increased metabolism of the oncoprotein fragment is uncertain.

The c-*erb*B-2 oncoprotein expression has previously been studied in a limited number of renal cell carcinomas. Herrera (1991), in an analysis on cystic renal disease using immunohistochemistry on formalin-fixed paraffin-embedded material, found overexpression of the c-*erb*B-2 oncoprotein in two out of five renal cell carcinomas. No correlation with disease stage or tumour grade was presented. Stumm et al (1996) found high levels of c-*erb*B-2 oncoprotein expression in 64% of fresh-frozen tumours using immunohistochemistry, but the relation to stage and grade was uncertain. In the present study, an inverse relation between tumour grade, disease stage, survival time and the serum level of c-*erbB*-2 oncoprotein was observed. Our results are in line with previous studies in colonic and ovarian cancer. Cohen et al (1989), using cell lines from colonic cancers, found lower c-*erb*B-2 expression in poorly differentiated tumours than in more differentiated tumours. McKenzie et al (1993) analysed soluble ectodomain of c-*erb*B-2 oncoprotein in ovarian cancer and found significantly lower levels in more advanced disease stages and a tendency towards lower levels in poorly differentiated tumours. In breast cancer, the c-*erb*B-2 oncogene expression was increased in more advanced disease stages and in poorly differentiated tumours (Slamon et al, 1987; Lupu et al, 1995), findings that are opposed to the results of the present study. Variable results have been presented in other studies of breast cancer in which expression was found to be at a higher frequency in ductal carcinoma in situ tumours than in invasive tumours (van de Vijver et al, 1988; Allred et al, 1992).

Univariate analysis of the prognostic value of the soluble ectodomain of c-*erb*B-2 in the present study shows that the level was inversely related to survival time. This result is opposed to the findings in breast cancer, in which overexpression of c-*erb*B-2 oncoprotein is a negative prognostic factor (Slamon et al, 1987; Tandon et al, 1989; Kandl et al, 1994). When prognosis was evaluated in a multivariate analysis in renal cell carcinoma, the strong predictors were stage and grade in accordance with earlier reports (Thrasher and Paulson, 1993), while c-*erb*B-2 oncoprotein lost its independent prognostic value.

In conclusion, an inverse relation between serum levels of the soluble ectodomain of c-*erb*B-2 oncoprotein and disease stage, tumour grade and survival time in renal cell carcinoma was found.

### ACKNOWLEDGEMENTS

This study was supported by grants from the Lions Cancer Research Foundation, the Medical Faculty, Umeå University, and the University Hospital, Umeå.

### REFERENCES

Allred DC, Clark GM, Molina R, Tandon AK, Schnitt SJ, Gilchrist KW, Osborne CK, Tormey DC and McGuire WL (1992) Overexpression of HER-2/*neu* and its relationship with other prognostic factors change during the progression of in situ to invasive breast cancer. *Hum Pathol* **23**: 974–979

Carney WP, Hamer PJ, Petit D, Retos C, Greene R, Zabrecky JR, McKenzie S, Hayes D, Kufe D, DeLellis R, Naber S and Wolfe H (1991) Detection and quantification of the human neu oncoprotein. *J Tumor Marker Oncol* **6**: 53–72

Cohen JA, Weiner DB, More KF, Kokai Y, Williams WV, Maguire HC, LiVolsi VA and Greene MI (1989) Expression pattern of the *neu* (NGL) gene-encoded growth factor receptor protein ($p185^{neu}$) in normal and transformed epithelial tissues of the digestive tract. *Oncogene* **4**: 81–88

© Cancer Research Campaign 1997

Coussens L, Yang-Feng TL, Liao Y-C, Chen E, Gray A, McGrath J, Seeburg PH, Libermann TA, Schlessinger J, Francke U, Levinson A and Ullrich A (1985) Tyrosine kinase receptor with extensive homology to EGF receptor shares chromosomal location with *neu* oncogene. *Science* **230**: 1132–1139

Freeman MR, Washecka R and Chung LWK (1989) Abberant expression of epidermal growth factor receptor and HER-2 (*erb*B-2) messenger RNAs in human renal cancers. *Cancer Res* **49**: 6221–6225

Herrera GA (1991) C-erb B-2 amplification in cystic renal disease. *Kidney International* **40**: 509–513

Kandl H, Seymour L and Bezwoda WR (1994) Soluble c-*erb*B-2 fragment in serum correlates with disease stage and predicts for shortened survival in patients with early-stage and advanced breast cancer. *Br J Cancer* **70**: 739–742

Leitzel K, Teramoto Y, Sampson E, Mauceri J, Langton BC, Demers L, Podczaski E, Harvey H, Shambaugh S, Volas G, Weaver S and Lipton A (1992) Elevated soluble c-*erb*B-2 antigen levels in the serum and effusions of a proportion of breast cancer patients. *J Clin Oncol* **10**: 1436–1443

Lupu R, Cardillo M, Harris L, Hijazi M and Rosenberg K (1995) Interaction between erbB-receptors and heregulin in breast cancer tumor progression and drug resistance. *Semin Cancer Biol* **6**: 135–145

McKenzie SJ, DeSombre KA, Bast BS, Hollis DR, Whitaker RS, Berchuck A, Boyer CM and Bast RC (1993) Serum levels of HER-2 *neu* (C-*erb*B-2) correlate with overexpression of p185$^{neu}$ in human ovarian cancer. *Cancer* **71**: 3942–3946

McKenzie SJ, Marks PJ, Lam T, Morgan J, Panicali DL, Trimpe KL and Carney WP (1989) Generation and characterization of monoclonal antibodies specific for the human *neu* oncogene product, p185. *Oncogene* **4**: 543–548

Mori S, Mori Y, Mukaiyama T, Yamada Y, Sonobe Y, Matsushita H, Sakamoto G, Akiyama T, Ogawa M, Shiraishi M, Toyoshima K and Yamamoto T (1990) *In vitro* and *in vivo* release of soluble *erbB*-2 protein from human carcinoma cells. *Jpn J Cancer Res* **81**: 489–494

Press MF, Corbon-Cardo C and Slamon DJ (1990) Expression of the HER-2/*neu* proto-oncogene in normal human adult and fetal tissues. Oncogene **5**: 953–962

Robson CJ, Churchill BM and Anderson W (1969) The results of radical nephrectomy for renal cell carcinoma. *J Urol* **101**: 297–301

Rotter M, Block T, Busch R, Thanner S and Höfler H (1992) Expression of HER-2/*neu* in renal-cell carcinoma. Correlation with histologic subtypes and differentiation. *Int J Cancer* **52**: 213–217

Schechter AL, Hung M-C, Vaidyanathan L, Weinberg RA, Yang-Feng TL, Francke U, Ullrich A and Coussens L (1985) The *neu* gene: an *erb*B-homologous gene distinct from and unlinked to the gene encoding the EGF receptor. *Science* **229**: 976–978

Skinner DG, Colvin RB, Vermillion CD, Pfister RC and Leadbetter WF (1971) Diagnosis and management of renal cell carcinoma. A clinical and pathologic study of 309 cases. *Cancer* **28**: 1165–1177

Slamon DJ, Clark GM, Wong SG, Levin WJ, Ullrich A and McGuire WL (1987) Human breast cancer: correlation of relapse and survival with amplification of the HER-2/*neu* oncogene. *Science* **235**: 177–182

Slamon DJ, Godolphin W, Jones LA, Holt JA, Wong SG, Keith DE, Levin WJ, Stuart SG, Udove J, Ullrich A and Press MF (1989) Studies of the HER-2/*neu* proto-oncogene in human breast and ovarian cancer. *Science* **244**: 707–712

Sprent P (1989) *Applied Nonparametric Statistical Methods*. Chapman & Hall: London

Stumm G, Eberwein S, Rostock-Wolf S, Stein H, Pomer S, Schlegel J and Waldherr R (1996) Concomitant overexpression of the EGFR and erbB-2 genes in renal cell carcinoma (RCC) is correlated with dedifferentiation and metastasis. *Int J Cancer* **69**: 17–22

Tandon AK, Clark GM, Chamness GC, Ullrich A and McGuire WL (1989) HER-2/neu oncogene protein and prognosis in breast cancer. *J Clin Oncol* **7**: 1120–1128

Thrasher JB and Paulson DF (1993) Prognostic factors in renal cell cancer. *Urol Clin North Am* **20**: 247–262

van de Vijver MJ, Peterse JL, Mooi WJ, Wisman P, Lomans J, Dalesio O and Nusse R (1988) *Neu*-protein overexpression in breast cancer. Association with comedo-type ductal carcinoma in situ and limited prognostic value in stage II breast cancer. *N Engl J Med* **319**: 1239–1245

Weidner U, Peter S, Strohmeyer T, Hussnätter R, Ackermann R and Sies H (1990) Inverse relationship of epidermal growth factor receptor and HER2/*neu* gene expression in human renal cell carcinoma. *Cancer Res* **50**: 4504–4509

Venter DJ, Tuzi NL, Kumar S and Gullik WJ (1987) Overexpression of the c-*erb*B-2 oncoprotein in human breast carcinomas: immunohistological assessment correlates with gene amplification. *Lancet* **2**: 69–72

Yao M, Shuin T, Misaki H and Kubota Y (1988) Enhanced expression of c-*myc* and epidermal growth factor receptor (C-*erb*B-1) genes in primary human renal cancer. *Cancer Res* **48**: 6753–6757

Yokota J, Yamamoto T, Toyoshima K, Terada M, Sugimura T, Battifora H and Cline MJ (1986) Amplification of c-*erb*B-2 oncogene in human adenocarcinomas in vivo. *Lancet* **1**: 765–767

Zabrecky JR, Lam T, McKenzie SJ and Carney W (1991) The extracellular domain of p185/*neu* is released from the surface of human breast carcinoma cells, SK-BR-3. *J Biol Chem* **266**: 1716–1720

© Cancer Research Campaign 1997